# Health professionals’ perceptions of colorectal cancer patients’ treatment burden and their supportive work to ameliorate the burden – a qualitative study

**DOI:** 10.1186/s12913-020-05520-y

**Published:** 2020-07-17

**Authors:** Anne Marie Lunde Husebø, Bjørg Karlsen, Sissel Eikeland Husebø

**Affiliations:** 1 Department of Gastrointestinal Surgery, Stavanger University Hospital, N-4019 Stavanger, Norway; 2 Department of Public Health, Faculty of Health Sciences, University of Stavanger, N-4036 Stavanger, Norway; 3 Department of Quality and Health Technology, Faculty of Health Sciences, University of Stavanger, N-4036 Stavanger, Norway; 4 Department of Surgery, Stavanger University Hospital, N-4011 Stavanger, Norway

**Keywords:** Colorectal cancer, Burden of treatment, Health professionals, Nursing, Self-management, Semi-structured interview, Support

## Abstract

**Background:**

Support is pivotal for patients in managing colorectal cancer treatment, as they might be overwhelmed by the burden of treatment. There is scarce knowledge regarding health professionals’ perceptions of colorectal cancer patients’ burdens and supportive needs. The study aims to describe health professionals’ perspectives on treatment burden among patients receiving curative surgical treatment for colorectal cancer during the hospital stay and how they support patients to ameliorate the burden.

**Methods:**

This study has a descriptive and explorative qualitative design, using semi-structured interviews with nine health professionals recruited from a gastrointestinal-surgery ward at a university hospital in Norway. Data were analysed by using systematic text condensation.

**Results:**

Data analysis identified the themes “capturing patients’ burdens of colorectal cancer treatment” and “health professionals’ support to ameliorate the burden”. Patients with colorectal cancer had to face burdens related to a challenging emotional situation, treatment complications and side effects, and an extensive need for information. A trusting patient-carer relationship was therefore perceived as the essence of health professionals’ support. Health professionals focused their support on safeguarding patients, motivating patients to self-manage, and involving family and peers as supporters. Patients’ journey characteristics and illness severity challenged health professionals’ supportive work.

**Conclusion:**

Support from health professionals includes providing patients emotional support and relevant treatment-related information and motivating patients for early post-surgical mobilisation. Health professionals should be aware of identifying colorectal cancer patients’ information needs according to the specific treatment stages, which may ameliorate the burden of colorectal cancer treatment and enable patients to self-manage.

## Background

Colorectal cancer (CRC) care requires a multi-discipline approach, engaging a diverse team of professionals to provide high-quality cancer care and support the patients throughout the treatment trajectory [1]. Social support is referred to as ‘*the emotionally sustaining qualities of relationships (e.g., a sense that one is loved, cared for, and listened to)*’ [[2], p. S51]. In the health care context, social support can be understood as the patient’s perception of the quality and availability of interactions with others, who provide practical assistance and informational and emotional support [3]. When perceived as positive, support from health professionals (HPs) can moderate or eliminate the negative consequences of treatment-related stress by making threatening experiences less overwhelming or by providing valuable resources for coping with self-management (e.g., self-monitoring of health status, finding and understanding medical information, managing therapeutic regimens, arranging medical appointments) [4]. Two main purposes of HPs’ self-management support have been outlined: ‘*to support and help patients manage their disease well, and to live well with the disease’* [[5] , p.246]. A study among colorectal cancer (CRC) patients established social support as an asset towards CRC patients’ quality of life, and that professional support predicts lower stress and higher resilience in the patients [6].

Colorectal cancer (CRC) is the third most common cancer globally [7]. In 2018, approximately 4428 Norwegians were diagnosed with CRC; this figure represents one of the world’s highest incidences of CRC [8]. In some countries, such as Norway, patients with CRC are enrolled in a cancer care pathway that runs from the time of diagnosis through treatment to follow-up in order to secure a seamless and effective diagnosis and treatment process [9]. Advances in CRC treatment involve fast-track surgery and enhanced recovery after surgery (ERAS), which aim at shortening the hospital stay and accelerating patient recovery [10]. The ERAS program entails different HPs engaging in patient education and counselling, optimization of the patient’s nutritional status and gastrointestinal functioning, providing pain relief, and facilitate early post-surgery mobilisation [11]. Treatment varies depending on the cancer stage at the time of diagnosis, but most patients receive surgical treatment. For some of the patients, the diagnosis of low, rectal cancer or surgical complications of anastomotic leakage results in the formation of a stoma, affecting the patients’ quality of life [12]. Patients with stage II or stage III are offered rectal cancer preoperative chemo-radiation as standard treatment [2], whereas patients diagnosed with CRC stage II or stage III often need adjuvant chemotherapy treatment post-surgery [13].

Accordingly, patients with CRC must deal with many dimensions of burden during their treatment trajectory and must balance their demands and their capacity to self-manage [14]. This burden of treatment can be described as the extra work that health professionals (HPs) delegate to patients to live well with their chronic illness [15, 16]. The transition from acute treatment to cancer survivorship at home or in the community setting is often poorly managed, and cancer survivors report that they often feel unprepared for the post-treatment period [17, 18]. CRC treatment guidelines do not provide recommendations for how to reduce and manage symptom burden and increase health-related quality of life [19]. Such recommendations could be useful to HPs in supporting CRC patients to live well and ameliorate the burden.

Evidence of CRC patients’ support needs from the patient’s perspective is well establish, indicating a vast physical, emotional and social illness and treatment burden. A review by Kotronoulas et al. [20] reported CRC patients’ support needs as mainly concerning information/education or patient-HPs communication issues, claiming that the relationship between patients with CRC and HPs may be fundamental to ensuring effective self-management tasks. Importantly, patients with CRC express requirements for support in terms of addressing their fear of cancer reoccurrence, receiving nutritional advice and information, and self-managing symptoms at home [21, 22]. CRC patients report HPs as their main important information source, especially during post-surgery treatment and survivorship stages, with physicians and nurses providing the most information [23]. Guassora, Jarlbaek and Thorsen [24] suggest that hospital HPs may play a key role in preparing cancer patients for the vulnerable transition between secondary and primary care by individualized coordination.

Earlier research is mainly concerned with HPs views on challenges or barriers to cancer care. In Banerjee et al. [25] oncology nurses identified areas of communication challenges as disclosing bad news to the patient, or not having enough time to prioritise emotional support. Among challenges relating to the patient-provider relationship in CRC care are supporting the patients during emotional distress and providing information adjusted to the patient’s needs [26]. HPs may also find it difficult to engage in conversation with CRC patients on sexuality [27] and lifestyle advice [28] due to lack of knowledge and access to a consultation system, fearing loss of a good patient-provider relation.

To date, HPs perspectives on CRC patients’ burdens and support needs during primary treatment are largely absent in research. More research is therefore needed to gain detailed insight into the supportive work of HPs caring for patients with CRC, to ameliorate the burden of treatment by facilitating recovery and strengthening their ability to self-manage their disease. Knowledge gained from this study may aid a deeper understanding of how HPs facilitate the CRC patient’s navigation through the illness and treatment trajectory while being hospitalized. It may also identify challenges to HPs supportive work towards patient’s information needs, management of emotional distress and a healthy recovery. Moreover, it may provide useful information to guide future interventions targeting a supportive CRC care. Hence, the aim of this study was to (i) explore treatment burden in CRC, and (ii) identify elements of HP support to amend the burden, addressing the research question:

How do HP describe aspects of treatment burden among patients receiving curative surgical treatment for CRC during hospital stay, and the support they provide to ameliorate the burden?

## Methods

### Design

This study applied an explorative and descriptive qualitative design [29] with individual interviews as the data collection method and systematic text condensation (STD) as the data analysis method [30]. A qualitative approach was deemed appropriate to explore HP experiences, meanings and perspectives, and identify themes and patterns to create a deeper understanding of CRC patients’ treatment burden during curative, surgical treatment [29].

This was a single-centre study conducted at one gastrointestinal surgical ward in a university hospital in an urban area in south western Norway. The hospital is providing health care to 18 municipalities with 350,000 citizens, and curatively treating approximately 170 patients for CRC each year.

### Participants

The sample consisted of HPs employed at the gastrointestinal-surgical ward, including registered nurses and gastrointestinal surgeons. They were recruited to the study by purposive expert sampling, a non-random technique typically used in qualitative research to identify information-rich individuals who are well-informed with the phenomenon under research [[31] , p. 2]. A staff nurse at the gastrointestinal-surgical ward identified and recruited HPs, who were willing and available to share their knowledge and clinical experience with CRC patient’s treatment burden relating to primary, surgical treatment. Recruitment was based on the following inclusion criteria: (1) authorised HP, (2) professional experience with treatment and care for patients with CRC, and (3) > 1-year employment at the current gastrointestinal-surgical ward. Of the HPs approached for participation, one HP withdrew the consent due to a changed work schedule including annual leave.

The nine HPs participating in the study comprised seven women and two men aged 22 to 52 years (mean age 33). Participants included seven RNs and two gastrointestinal surgeons with vast professional experience from gastrointestinal surgical cancer treatment and nursing care (range: 1- > 10 years). Two of the participants had over 10 years’ experience, two 4–7 years’ experience, and five 1–3 years’ experience. The participants were involved in patient treatment and care during diagnosis and pre, −peri,- and post-surgical stages of the hospital treatment trajectory, involving diagnostic procedures, cancer care coordination, treatment information, CRC nursing care, CRC surgical procedures, post-surgical recovery and discharge.

### Data collection

Data were collected in 2017 using a semi-structured interview guide to organise the interviews [29]. The development of the interview guide was based on earlier research on support for the self-management of long-term illness and theory on burden of treatment [16]. The guide consisted of 21 questions comprising two main themes: 1) how the participants perceived CRC patients’ treatment burden pre- and post-hospital stay and 2) how the participants perceived their efforts to provide adequate information and support (Table 1). The interviewer’s version of the guide included additional questions to facilitate responses whenever required. The guide was peer-reviewed by two researchers experienced with semi-structured interviews as a data collection method. At the beginning of the interviews, the participant was provided with information on the interviewer’s clinical and academic background and on the aim of the study. At the end of each interview, participants were asked to supplement their responses to ensure adequate representation of their perceptions. The interviews took place in an appropriate room at the hospital, with only the participant and the researcher present. The first author (AMLH) performed the interviews, which lasted between 35 and 90 min. The interviewer kept an interview log to capture any new issues relevant to the study aim brought up by the interviewee. Such themes were included in the interview guide for further investigation in the following interviews (e.g., the patient diary). Serial interviews were not conducted due to the chosen research design and the purpose of the study [32]. Data sampling continued until data redundancy was reached, confirmed by no new information emerging from the interviews, and a rich information power was secured [33]. None of the participants was deemed as atypical for the group of HPs working at the gastrointestinal-surgical ward, based on confirmation of interview themes, and similar professional background within professions [33]. All interviews were audiotaped and transcribed verbatim.

**Table 1:** Interview guide

Theme	Main question	Additional question
Perceptions of CRC patients’ treatment burden in the treatment trajectory during hospitalisation	From your point of view, what challenges do patients face? In what ways do you expect the patient to self-manage following surgery? What factors come into play concerning the patient’s ability to self-manage? What information needs do patients and their families usually have?	What are the biggest challenges? Do you find that they follow your advice? Can you give any examples? How do, e.g., age, gender, and family situation matter? What are their information needs -Pre-surgery-Post-surgery-At discharge?
Perceptions of provision of support	How do you prepare the patient to cope with self-management following surgery? What approaches are being taken to strengthen the patient’s self-management following discharge? What is the greatest challenge in providing the patient with information?	What is your specific responsibility towards the patient’s recovery process? What information routines are used at discharge? Who is responsible for informing the patient at discharge? What specific information is important to give patients at discharge? How do you involve the patient’s family? How do patients and their family receive the information you give them?

### Ethical considerations

The National Committee for Research Ethics in Social Sciences and the Humanities (No. 2017/284) and the hospital’s research department approved the study. All participants provided informed written consent before the individual interviews and were guaranteed confidentiality and the right to withdraw from the study at any time.

### Data analysis

Transcripts were thematically analysed using QRS International’s software programme QSR International’s NVivo 12 [34] to systemise and code the data. The STC [30] is a pragmatic strategy for thematic analysis of the meaning and content of data across cases. All authors contributed to the analysis. They are all female, registered nurses, holds a PhD in health sciences and are experienced with qualitative research methods. At the time of the study, the first author was a Postdoctoral Fellow at a university hospital, while the second and third authors worked as academics.

The analysis process consisted of four steps. In the first step, the transcripts were divided between the authors and read through multiple times. Then, all three authors independently performed the analysis by employing a comprehensive read-through of the transcripts, resulting in three preliminary themes derived from the data: ‘perceived patient challenges’, ‘perceptions of CRC patients’ need for support’, and ‘being an information provider’. The second step consisted of coding through deduction of the meaning units within each preliminary theme by identifying and negotiating text fragments containing information about the research question. In the third step, the meaning units were compared and coded based on similarities within and differences between the themes. Finally, in step 4, themes were then formed by grouping codes and rechecked across the entire data set. The themes were refined and discussed between the authors until a consensus was reached. Two main themes with different sub-themes were identified (see Table 2).

**Table 2:** Examples of the analysis process

STEP 1 Preliminary themes	STEP 2 Meaning units (an example)	STEP 3 Sub-categories	STEP 4 Main categories
Perceived patient challenges	They get very upset and devastated. It is a life crisis in a way … it is heart-breaking to watch their reactions and difficult to know how to help them in the best possible way. You really can’t tell them everything will be fine because sometimes it isn’t. (Nurse, interview 1) Then they get retention, or they get anastomosis leakage, or they get facie rupture. Then, it will be a longer course … then, I see the crisis approaching. This is the biggest challenge. (Nurse, interview 5) They are very curious about things. They not only ask the doctor who does the rounds, but they ask the auxiliary nurses, the evening shift nurses who do not know the patient from the day shift. Then, you lose information, and there will be misunderstandings. It is very challenging and frustrating for the patient. (Surgeon, interview 3)	Emotional responses burdening patients Being troubled by treatment complications and side effects The patient’s unmet needs for information increases the burden	Capturing patients’ burden of CRC treatment
Perceptions of CRC patients’ need for support	If they feel safe, they can handle almost anything … you need to make things look a bit less serious. (Nurse, interview 2) Our wish is that they learn as much as possible. We try to give them tools for coping so they feel ready to go home. (HP5) They (the patient organisation) come here a lot. Actually, not everyone wants to meet with them. They (the patients) are in a post-surgical stage, and there are so many new things with the ostomy they have to take in. They get a card and can contact them if they want to. They will always be there for them. (Nurse, interview 8) Sometimes, I get all choked up and teary-eyed just by thinking of the patient’s situation. It is a difficult challenge because it’s cancer. (Surgeon, interview 3)	Creating safe environments Motivating and supporting patient self-management Facilitating contact with family and peer support Facing challenges in providing sufficient support	Health professionals’ supportive work

Trustworthiness was achieved through investigator triangulation in the analysis to obtain optimal inter-subjectivity [29]. We also used the criteria of credibility, dependability and transferability to ensure the accuracy of the research [35]. To reinforce the credibility of the data collection, the same author (AMLH) conducted all interviews. Data coding was first performed by the first author, and the coding was then negotiated in an analysis workshop involving all authors. In addition, all three authors discussed the findings and interpretations of the subgroups and main categories until agreement was reached. This process is referred to by Lincoln & Guba [35] as peer checking, which is a substitute for interviewees’ checking of transcripts and has been found to be a valid means to increase the credibility of findings. The dependability of the study was ensured by using same interview guide for all interviews, audio taping and transcribing the interviews verbatim, and importing them into QRS International’s NVivo 11 software. Transparency was achieved by providing the reader with a display of the data analysis steps (Table 2). The transferability of our findings to another context was enhanced by using illustrative quotations from the data. To achieve comprehensive and explicit reporting of the study, reporting of study findings was conducted in line with the COREQ (COnsolidated criteria for REporting Qualitative Research) Checklist [36].

## Results

The analysis resulted in the identification of two themes: “capturing patients’ burden of CRC treatment and “HPs’ support to ameliorate the burden”. The first theme is based on three sub-themes and the second on four sub-themes (see Table 2). In the following text, the contents of the themes and sub-themes are described in detail with quotations from the participants.

### Capturing patients’ burden of colorectal cancer treatment

This theme emerged from the HPs’ description of patients’ burden of CRC treatment and how they tried to capture these burdens. The theme was characterised by the following three sub-themes: (1) emotional responses burdening patients, (2) being troubled by treatment complications and side effects, and (3) the patient’s unmet need for information increasing the burden.

### Emotional responses burdening patients

The majority of the participants described dealing with patients with a range of emotional responses. Sometimes, patients came in with a stomach-ache and constipation in need of emergency surgery, leaving less time for the HPs to inform the patient. Those patients often responded with mental shock and crisis when realising it was cancer and appeared to the HPs as being in loss of control over their life. Some of the participants perceived that patients did not have time to prepare mentally for the surgical procedure, and even though they were reassured of a successful surgical outcome, patients did appear to feel safe.

*“They get very upset and devastated. It is a life crisis in a way … it is heart-breaking to watch their reactions and difficult to know how to help them in the best way possible. You really can’t tell them that everything will be fine because sometimes it isn’t”* (Nurse, interview 1).

The participants said that some patients were overwhelmed by everything that was going on during the admission and showed clear signs of discomfort, which resulted in inactivity and a lack of motivation for recovery. In some cases, patients expressed the desire to stay in the hospital as long as possible. In the respondents’ opinion, patients’ responses to the short treatment time and to early discharge from the hospital depended on HPs’ efforts to safeguard patients’ need for information and support.

*“It is the whole situation … they have had bowel surgery, and they might think that going home represents a huge challenge … and that we sort of ‘kick them out’. As long as we explain that this is normal … you can do it, just take it easy. Going home to your own bed and eating your own food are often the best things for a healthy appetite and a good night’s sleep”* (Nurse, interview 7).

Many participants indicated that enrolment in an efficient cancer care pathway gave patients little time to absorb and react to the fact that they had a serious illness needing treatment*.* In addition, a short hospital stay meant leaving the hospital without all the answers needed.

*“Everything has to happen so fast. As a patient, you are diagnosed and treated within two weeks time following your first suspicion that something is wrong. It’s a good thing but also a challenge … I don’t know … I think patients feel they are in a vacuum. In addition, the patients have to wait for further tests and treatment … can it be treated or not? There are a lot of aspects here … I really don’t know what’s the worst!”* (Surgeon, interview 9).

In some cases, patients took on their next-of-kin’s worries and made them their own, which added to the emotional burden. This became particularly obvious if the patients were parents of small children, as patients feared how the children would be affected by having a seriously ill parent.

*“‘What will happen if I have metastatic cancer?’ the patient asks. The younger patients with small children … if there are children involved, the challenges are immense”* (Nurse, interview 6).

According to the participants, the waiting time between surgery and the first post-surgery appointment at the outpatient clinic constituted an emotional burden for patients. At the appointment, patients are informed by the physician of any detected metastasis or need for further treatment*.*

*“Of course, it’s nerve-wracking because you will never know one hundred percent before the first appointment several weeks after surgery when they have the biopsy results ready if you need more treatment. Our patients are positive … hope they are cured and leave for home in good spirits … but of course with a touch of excitement. There will always be something unanswered”* (Nurse, interview 2).

The participants indicated that being enrolled in a 5-year follow-up programme was an asset and a necessity, but they also viewed it as a treatment burden. On one hand, they emphasised that it could be a relief for patients to be monitored as patients knew that health care services were in control of a potential cancer relapse. On the other hand, they expressed it as a long-lasting burden to the patient, not knowing for years if they were really cancer free.

### Being troubled by treatment complications and side effects

Many participants expressed that patients were burdened by surgical complications, primarily wound ruptures, intestinal leakage, surgical wound infections and ventricle retention. They described these complications as severely challenging for patients and noted in some cases, it prolongs the hospital stay and recovery.

“*Then, they get retention, or they get anastomosis leakage, or they get facie rupture. Then, it will be a longer course … then, I see the crisis approaching! This is the biggest challenge”* (Nurse, interview 5).

In the participants’ views, treatment side effects involving post-surgical pain, nausea and vomiting due to reduced bowel movement represented a burden to patients. They reported that many patients were striving to regain normal bowel function and a healthy appetite. In some cases, complications and side effects led to immobilisation among patients who had a hard time getting back on their feet and being active. The participants expressed this as a challenge for recovery and the avoidance of further complications.

The majority of the HPs spoke of having an ostomy as a major treatment burden. They expressed the physical, emotional, and relational burden that patients had to endure and noted that the fear of having an ostomy often exceeded patients’ fear of the surgery itself.

*“The last thing in the world! … they would do anything to avoid it (the ostomy). Most of them think it’s horrible, and many can’t even bear to look at their stomach. It is a big challenge for them”* (Surgeon, interview 3).

Many participants were concerned with how an ostomy often represented a huge strain for patients, as it affected body functioning and patients’ self-image and represented a threat to their sexual health. They stated that some patients viewed the ostomy as a stigma and were shameful of it. Some of the participants expressed concerns that information on sexual health following colorectal surgery was rarely communicated to patients. Although poor sexual function was highlighted by the participants as a well-known side effect from rectal amputation, they called for a greater focus on sexual health as part of post-surgical information and a need for increased knowledge and competency among staff.

*“Getting an ostomy is a huge intervention disturbing the self-image. It is important to be open about it, especially when it comes to sex”* (Nurse, interview 8).

### Patients’ unmet needs for information increasing the burden

Several participants talked about how patients often expressed a need for exhaustive information. The fast-track treatment procedures not only left patients little time to recover at their own speed but also made it challenging for participants to provide patients with information and support. In their view, patients’ unmet information needs led to an ongoing information hunt that involved different HPs, who might give ambiguous answers.

*“They are very curious about things. They not only ask the doctor who does the rounds, but they ask the auxiliary nurses, the evening shift nurses who do not know the patient from the day shift. Then, you lose information, and there will be misunderstandings. It is very challenging and frustrating for the patient*” (Surgeon, interview 3).

Patients’ need for information, as experienced by the participants, was mostly related to the surgical procedures, but they also expressed a need for answers to questions such as “What will happen next? What will happen to me?” They were eager to get back to a normal life and continue with their lives, and they sought information about physical activity, diet, sexual function and daily life activities.

“*In a few days’ time, you are treated and you have the surgery. It happens quickly. There are many who have not fully realised that something was wrong in the first place. A lot of them respond by seeking all the information they can find and are caught up in it”* (Nurse, interview 4).

It was clear to the respondents that uncertainty regarding discharge and self-management constituted a burden for patients. Although the discharge routines involved extensive verbal and written information, the participants feared that some patients were left to themselves with many unanswered questions, unmet information needs and nobody to turn to.

*“Many patients feel that everything stands still when they are here, so then, it starts when they come home … the crisis may hit. Then, there are no doctors’ rounds. Perhaps the ostomy was not as productive as the day before. They do not quite have the safety net of professionals like they had here”* (Nurse, interview 5).

### Health professionals’ supportive work to ameliorate the burden

The second theme describes the participants’ experiences of their supportive work to ameliorate the burden. This theme contains four sub-themes: (1) creating safe environments, (2) motivating and supporting patient self-management, (3) facilitating contact with family and peer support and (4) facing difficulties in providing sufficient support.

### Creating safe environments

The participants were concerned with making patients and their families feel safe by reassuring them that they were safe and receiving the best treatment. This meant being there for patients and providing information and support. The HPs viewed trust as the essence of a safe environment. A safe environment involved the coordinator of the cancer care pathway, who closely followed up with patients regarding appointments and tests, and the surgeons and nurses, who provided thorough information on surgical procedures, offered emotional support, and reassured patients that they would cope at home.

*“You have to make it clear to them that life goes on right after the surgery; time does not stand still while they are here. A lot of them feel that way, thinking life goes on when I come home. It is as if they are using a stand-by button. Then, you have the opposite ones … those who are being careless. Then, I need to hold them back a little. It is important to know who you are dealing with … talk to the family and get to know the patient”* (Nurse, interview 5).

### Motivating and supporting patient self-management

An important goal of the HPs’ support, as expressed by all participants, was to facilitate early post-surgical mobilisation. The participants explained that colorectal surgery is prone to complications, and early mobilisation of the patient was stressed as crucial to avoid complications and a prolonged hospital stay. Some participants explained how they needed to balance the intensity of the motivational initiative and were aware that patients might feel a pressure to perform. They were concerned with making patients responsible for their own recovery and helping them to regain control and restore normality. They expected that patients would respond to their support by adhering to advice on activity, diet and medications.

*“We expect them to be up and about, start to eat and drink a little, stressing the need to get out of bed to avoid complications but also to make them get back on their feet and recover from the surgery. Therefore, we expect them to listen to what we suggest, and usually, they do fine. In a way, they are prepared for it because we tell them prior to surgery that there are a lot less complications if they quickly get back on their feet”* (Nurse, interview 1).

Most of the participants also said that they motivated patients to self-manage and to cope with their new life situation by increasing their self-management and coping skills. In particular, patients who received an ostomy needed support in becoming motivated to adjust to changed bodily functions and a new body image. HPs support entailed a careful approach to teaching patients how to take care of the ostomy (support that involved an ostomy care nurse) as well as ordering ostomy equipment for the patient to use after discharge.

*“Our wish is that they learn as much as possible. We try to give them tools for coping so they feel ready to go home ( …*) *I think that sometimes we impose particular skills on them, you need to learn this and that … you must have changed your ostomy bag a certain amount of times before you go home. I think I manage quite well to accept the times when they* (the patients) *don’t want to be responsible. It’s their way of coping”* (Nurse, interview 5).

Another incentive to motivate patients’ self-management expressed by the participants was a patient diary that patients received at admission on the ward. The HPs encouraged patients to record how they were doing post-surgery, including their daily self-management activities, such as hours spent out of bed, dietary activities, bowel movements and pain levels.

*“There is a big difference between those who use the tools we give them and those who don’t. We have a diary for them to fill out. I don’t know, but those who actively use it, they take responsibility for their own treatment in a way, and they get much more out of it and get out of hospital faster”* (Nurse, interview 4).

### Facilitating contact with family and peer support

The participants emphasised the importance of not ignoring patients’ relatives but inviting them in as patient supporters*.* It was important to the participants to create a ward environment that included the patient’s family. Efforts to involve the family depended on family relations, whether and how often family members would visit the patient on the ward, and whether the patient consented to the hospital making contact with the family. Some family members initiated their participation in treatment meetings, which the participants viewed as important given the high information intake.

*Many times, the next-of-kin will take part in treatment meetings. Then, two sets of ears listen to what the plan is … if one of them picks up on 10% of the information and the other one 10%, they have 20% altogether!”* (Nurse, interview 2).

In addition, some participants expressed that illness severity sometimes determined how they approached the families, and they explained how supporting the patient also meant supporting the family.

*“Someone needs to be in control, you know. Many of them receive continuous information when they visit, and some ask for a meeting with the nurse and the doctor. It has a lot to do with the severity of the illness. It is, of course, the patient who is ill, but cancer affects the whole family. You can’t forget about the next-of-kin because they are such great supporters. If the next-of-kin are doing well, the patient is doing even better”* (Surgeon, interview 3).

Some participants did not perceive family members as supportive. Although they valued the families as important supporters of patients’ recovery and encouraged them to take part in the support, they also described situations in which family members had the opposite effect on the patient’s motivation. The participants described how husbands or wives were reluctant to take the patient home, or they took over the patient’s self-management tasks. Moreover, for some patients, worried next-of-kin might even prolong the hospital stay. The participants described family members as uncertain and anxious at the thought of taking responsibility for the patient’s recovery. Although the participants expressed their understanding of next-of-kin’s actions as a crisis reaction, they sometimes perceived the actions as non-supportive and disruptive for the patient’s self-esteem and courage.

*“The patients who have a lot of family around get affected by them. As soon as they get rid of their own worries, they take on their family’s worries. For instance, the patient may have recovered fine, he is up and about, eating and the bowels are working again. Then, the family comes to visit, and the patient suddenly appears much worse and can’t go home. The hospital stay will then be prolonged because of the family’s worries … even if the patient would have managed fine at home. I don’t think most people realise how healing it can be to come home to familiar surroundings!”* (Nurse, interview 7).

All participants talked about how peer support contributed to ostomy patients’ self-care. Peers from a patient organisation visited the ward regularly, and the HPs explained how they made conscious use of the peer support by informing patients of their peers’ presence on the ward and facilitating contact between them. The peers would offer patients advice on ostomy equipment and care based on their own experience from living with an ostomy. Two of the participants noted that not all patients were ready to meet with a peer and talk about the ostomy.

*“They* (the patient organisation) *come here a lot. Actually, not everyone wants to meet with them though … they* (the patients) *are in a post-surgical stage, and there are so many new things with the ostomy they have to take in … it all happens so fast. They get a card and can contact them if they want to. They will always be there for them”* (Nurse, interview 8).

### Facing challenges in providing sufficient support

The participants described how they faced challenges in their effort to support and motivate patients. They described a difference in their supportive work with patients who had a scheduled admission and those who were emergency admissions. With scheduled admissions, the HPs had more time to inform and prepare the patient before surgery. In contrast, one of the respondents explained how emergency admissions challenged the HPs’ routines for preparing the patient.

*“Everything goes fast from the moment they get the CT result until they lay on the table in the operating theatre. Therefore, you do the most important things, and there is not enough time to go through everything. Then, again, you go through everything, but they are in shock and will not remember what you said anyhow. I don’t know what the biggest challenge is; that we don’t get to give them all of the information or the fact that they feel that there is too much information”* (Nurse, interview 2).

Being the messenger of bad news was a particular challenge experienced by some of the respondents. Information on diagnosis or prognosis, which became a burden to the patient, or information on an unexpected treatment complication was perceived as difficult to convey. Two of the respondents described this responsibility as finding a balance between being honest and being gentle when approaching the patient because they did not want to scare patients but still wanted to be realistic. When receiving the information, some patients reacted with anger and sadness, while others became apathetic, which the participants sometimes found hard to handle.

Some participants were also challenged in their informational work by not being able to provide patients with the information they required because they were waiting for the final test results, which might determine the future treatment course and prognosis. In most cases, patients had to leave the hospital without this crucial knowledge.

*“It is really difficult to plan for the follow-up while the patient is still in the hospital. The patients are very eager to know about this. What will happen next? Do I need more treatment? Do I need chemotherapy? We don’t know that until the test result is back from histology. So, they are sent home without a final plan for follow-up, and to some, this is difficult to understand”* (Surgeon, interview 9).

## Discussion

This paper reports findings from a qualitative study to enhance the understanding of the supportive work of HPs caring for patients with CRC to ameliorate the burden of treatment. The findings reveal a comprehensive picture of how HPs perceive CRC patients’ burden of treatment and how they reflect on their supportive work to ameliorate the burden. In the next section, the findings will be discussed according to these two aspects.

### The burdens of surgical treatment of colorectal cancer

Our findings suggest different aspects of burdens of treatment among CRC patients described by HPs. Not surprisingly, the emotional burden was identified as a vast burden to endure cancer treatment. According to the participants, this burden originated in the patient’s mental preparation level, the characteristics of the course of treatment and the family situation. These findings are congruent with those of Browne et al. [37], who found that the emotional reactions of patients with CRC involve uncertainty related to the surgical procedures, the surgery outcomes and worries about the future. From the HPs perspective, a swift treatment pathway with a short time frame from diagnosis to treatment may leave patients in a void. Norwegian cancer care pathways for CRC have an ideal short timeframe from diagnosing symptoms to initiating treatment with the aim to ensure high-quality cancer care [9]. Although waiting time is associated with significant concerns for cancer patients and delay of treatment may have an especially substantial psychological impact on patients [38], it is likely some patients experience a fast cancer care trajectory as stressful and challenging to keep up with. A structured treatment pathway may represent a treatment burden to cancer patients, requiring the patients to adapt to the pace of the treatment trajectory and set aside the demands of everyday life [15].

The findings also show that balancing an extensive need for information and feelings of being overwhelmed by a great information load may challenge patients as an active part of recovery and rehabilitation following surgical treatment of CRC. The participants were aware of the burden faced by many patients who had to find and understand information during their hospital stay, and participants indicated that the severity of the situation may hinder patients’ ability to process and make use of the information. Finding and understanding relevant information are included in the workload cancer patients need to actively manage in order to live well with their condition and treatments [15, 39]. Their information needs should therefore be identified and met according to the specific treatment stages [40].

The participants expressed concerns for patients who received an ostomy, noting that the ostomy represents substantial distress and a major treatment burden for patients. Managing an ostomy is among the consequences of CRC treatment adding to the complexity of the disease and requiring a proactive patient role [14]. According to Näsvall et al. [41], in rectal cancer patients, an ostomy is found to affect quality of life through decreased physical and mental health functioning, increased fatigue and a distorted body image. In particular, the early period after surgery can represent a vulnerable time, as faecal leakage and odour and noise from the ostomy bag are most prevalent [42]. Moreover, study findings highlight the need for attention to patients’ sexual health as part of HPs support following CRC surgery, and in particular male colon and rectal cancer patients report higher levels of sexual dysfunction and less sexual activity compared to a healthy population [43, 44].

### Supportive work to ameliorate treatment burden

Our findings show that HPs provided support and information throughout the cancer care pathway but were challenged by the CRC treatment programme, which included fast-track surgery and ERAS, in meeting patients’ extensive and ongoing need for relevant and sufficient information and support. Cancer patients’ support needs vary across the cancer continuum, and the most sought-after informational support in all phases is information about treatment. It is equally important in the pre-habilitation stage and the rehabilitation stage of the CRC care pathway to intervene to support patients’ recovery process and to reduce the treatment burden [40]. HPs’ actions of psychosocial and informational support can reassure patients and offer them strategies to cope with a stressful situation. According to Appleton et al. [45], positive interactions with HPs may increase cancer patients’ confidence in the treatment, strengthen their coping skills and help them adapt more easily to the treatment environment.

The current study demonstrates that special attention from HPs is needed for family members who struggle with sharing responsibility for the patient’s recovery. In contemporary health care services, there is an increasing expectation of patient and family engagement in the management of long-term illness [14]. HPs are encouraged to employ a whole-systems framework in their cancer care, prioritising a relational approach to cancer as patients and their families move through the cancer care pathway [46].

Establishing a trusting patient-carer relationship was expressed as the essence of HPs’ support for the patient’s burden of treatment in our study. A therapeutic supporting relationship is fostered through mutual trust, being sensitive to oneself and others, and meeting the patient’s needs through skills and knowledge [47]. Although they faced challenges of a having tight timeframe for support provision and having to deal with emotionally stressful situations, it was important for the HPs to build trust throughout the patient journey, characterised by availability, honest information and reassurance, and care for both the patient and the family. In a prospective study among patients with cancer and physicians, patient trust in the physician was related to a deteriorated physical condition mediated by patient enablement [48]. In light of our findings on support from HPs, enabling patients with CRC to understand and cope with the situation through informational and empathetic support holds the potential to affect the patients’ trust and quality of life.

Many of the participants emphasised treatment complications as a potential treatment burden for patients that led to a prolonged hospital stay and recovery. Thus, motivation to prepare patients for early mobilisation post-surgery was of utmost importance. Similarly, the ERAS guidelines suggest that HPs’ supportive work lasts for the entire patient journey to secure a complication-free and rapid patient journey [10]. Supporting the patient from preadmission (through preoperative information on pre-surgery lifestyle alterations and nutritional support) to the postoperative phase (focusing on mobilisation, return of gut function, control of pain and nausea and increased energy levels) is therefore of greatest importance.

According to the respondents in our study, HPs support and care may represent a security net for patients during hospitalisation when HPs are there for patients and provide information and a foundation for optimal rehabilitation after hospitalisation. This is important because many patients have less contact with specialist HPs following discharge and less access to the information they acquire to cope with the situation [21]. Research by Browne et al. [37] finds that information on surgical outcomes, what symptoms to monitor and how to deal with them and further plans for follow-up are crucial parts of HP informational support. Moreover, time spent counselling patients with CRC while they are hospitalised is found to shorten the hospital stay and improve postoperative physical and psychological quality of life [49]. Many different health care professions are involved in the care pathway of patients with CRC; thus, a holistic approach can improve treatment outcomes and the well-being of the patient and family.

As pointed out by the participants, the rapidness of the patient journey may be in conflict with HPs’ intentions to fulfil their obligations regarding patients’ right to information, thus hindering patient engagement. On the other hand, HPs provided continuous support on practical, motivational and emotional levels with the intention of ensuring an early recovery. One could argue that HPs support during CRC treatment is intended for patients to manage their condition and situation well and not to live well with the condition over time; this approach to support could be considered narrow [5]. A narrow approach comprises support strategies such as didactic education and persuasive motivation to aid condition control and patient compliance, with less attention paid to patient involvement [5].

### Limitations

The study has some limitations. The adequacy of the sample size was continuously evaluated during data collection, following Malterud et al.’s model of information power [50]. Information power was secured by a narrow study aim; participants who possessed broad experience with CRC treatment and care; interviews characterized by strong dialogue; a theoretical perspective on support and treatment burden supporting the study, and finally performance of an appropriate, thematic analysis [50]. Thus, we believe the sample provides enough information power to answer the research question and generate new knowledge. Hence, studying a phenomenon in depth and detail does not require large study samples [33]. Nevertheless, recruiting from more than one study site might have offered a more diverse participant sample, improving the generalisability and external validity of the findings, and made them more easily transferred to practice [51]. Also, while the study employed purposive sampling, the sample was unbalanced regarding the participants’ profession and gender, which may have created a bias. Including more male HPs and of other cancer care professions (e.g. physiotherapist, nutritionist, oncologist) may have added more depth to the findings.

Transcripts were not returned to the participants for comments, which may have increased the credibility of results [52].

## Conclusions

This is the first study to explore HPs’ experiences of burden among patients with CRC receiving curative surgical treatment, and their supportive approaches towards ameliorating the burden. The findings indicate that HPs acknowledge several aspects of burdens among CRC patients. The most pressing burdens are related to patients’ emotional reactions to cancer and its treatment and the need to balance information needs and information overload. Moreover, the study highlights the fact that these burdens can be ameliorated by supporting patients throughout the cancer care pathway and providing patients with relevant information, increasing their motivation for early post-surgical mobilisation, and offering emotional support. Our findings support the notion that assessing CRC patients’ support needs pre-surgery, closely following up on patients’ treatment burden and self-management achievements post-surgery, and actively using these achievements in motivational support are fundamental to the provision of quality of care for this patient group. It is also crucial to map patients’ social network resources and including them in patient care. However, further research is required before definite conclusions can be drawn about the dynamics of the CRC burden of pre- and post-surgery treatment and HPs supportive work. Such research may include the perspectives of CRC patients and their relatives on treatment burden support and how treatment burden may influence quality of life. Future health care interventions should be tailored specifically to capture the treatment burden of patients with CRC, to meet the supportive needs of patients and their families, and to ensure a safe transition from the hospital to the home.

## Data Availability

Datasets, containing deidentified data used and/or analysed during the current study will be available from the corresponding author on reasonable request.

## Abbreviations

CRC: Colorectal cancer
COREQ: COnsolidated criteria for REporting Qualitative research
ERAS: Early recovery after surgery
GP: General practitioner
HPs: Health professionals
